# Dietary habits in adolescence and midlife and risk of breast cancer in older women

**DOI:** 10.1371/journal.pone.0198017

**Published:** 2018-05-30

**Authors:** Alfheidur Haraldsdottir, Johanna E. Torfadottir, Unnur A. Valdimarsdottir, Hans-Olov Adami, Thor Aspelund, Laufey Tryggvadottir, Marianna Thordardottir, Bryndis E. Birgisdottir, Tamara B. Harris, Lenore J. Launer, Vilmundur Gudnason, Laufey Steingrimsdottir

**Affiliations:** 1 Faculty of Food Science and Human Nutrition, University of Iceland, Reykjavik, Iceland; 2 Centre of Public Health Sciences, Faculty of Medicine, University of Iceland, Reykjavik, Iceland; 3 Department of Epidemiology, Harvard T.H Chan School of Public Health, Boston, Massachusetts, United States of America; 4 Department of Medical Epidemiology and Biostatistics, Karolinska Institutet, Stockholm, Sweden; 5 The Icelandic Cancer Registry, Reykjavik, Iceland; 6 Faculty of Medicine, University of Iceland, Reykjavik, Iceland; 7 Unit for Nutrition Research, University of Iceland and Landspitali National University Hospital, Reykjavik, Iceland; 8 Laboratory of Epidemiology and Population Sciences, Intramural Research Program, National Institute on Aging, Bethesda, Maryland, United States of America; 9 The Icelandic Heart Association, Kopavogur, Iceland; University of Zurich, SWITZERLAND

## Abstract

Recent studies indicate that lifestyle factors in early life affect breast cancer risk. We therefore explored the association of high consumption of meat, milk, and whole grain products in adolescence and midlife, on breast cancer risk. We used data from the population based AGES-Reykjavik cohort (2002–2006), where 3,326 women with a mean age of 77 years (SD 6.0) participated. For food items and principal component derived dietary patterns we used Cox proportional models to calculate multivariate hazard ratios (HR) with 95% confidence intervals (95% CI). During a mean follow-up of 8.8 years, 97 women were diagnosed with breast cancer. For both adolescence and midlife, daily consumption of rye bread was positively associated with breast cancer (HR 1.7, 95% CI 1.1–2.6 and HR 1.8, 95% CI 1.1–2.9, respectively). In contrast, persistent high consumption of oatmeal was negatively associated with breast cancer (0.4, 95% CI 0.2–0.9). No association was found for other food items or dietary patterns that included rye bread. High rye bread consumption in adolescence and midlife may increase risk of late-life breast cancer whilst persistent consumption of oatmeal may reduce the risk.

## Introduction

During adolescence the female mammary tissue undergoes extensive modeling or re-modeling. Consequently, researchers have hypothesized that breast tissue may be particularly susceptible for initiation of breast tumors during this period [1, 2]. There is increasing evidence on the importance of adult midlife diet and risk of breast cancer [3] while available studies on the impact of diet during adolescence on breast cancer risk are scarce and somewhat inconsistent [4]. Studying early life diet can be challenging due to potential misclassification bias, the need for a long follow-up and limited variation in food intake between participants. Interestingly, there was considerable variability in dietary habits between residency areas in Iceland in early and mid 20^th^ century due to relative isolation of regions and differences in food access. This variation was observed to be strongest for the most common food items consumed at that time, or meat, rye, milk products, fish and fish oil [5]. These products are also of interest in this context because of their diverse bioactive compounds [6, 7] as well as their wide-spread use in modern Western diets.

In the Nurses' Health Study, women in the highest quintile of red meat consumption in adolescence had significantly higher risk of breast cancer than women in the lowest quintile [8–10]. A recent meta-analysis of 14 prospective studies [11] on adult red- and processed meat consumption reported a slightly increased breast cancer risk and similar results were observed in the NIH-AARP Diet and Health Study [12]. In contrast, no association with either childhood or adult milk consumption and risk of breast cancer has been found [13–18]. High total dietary fiber intake in early adulthood was associated with significantly lower breast cancer risk [19], as was total high adolescent consumption of lignans [20], a common phytoestrogen commonly found in whole grains [21]. High consumption whole grain food intake in adolescence and early adulthood was associated with lower risk of premenopausal breast cancer risk but not with post-menopausal risk [22]. Available studies on total adult whole grain consumption and breast cancer risk have either suggested a negative [22–24] or no association [25, 26].

For this study we used data from the population based AGES-Reykjavik Study, which is derived from a population with considerable variation in dietary habits in adolescence. Using the same cohort, we have previously observed a preventive role of very high fish intake in adolescence and midlife for breast cancer [27] and also the importance of earlier diet for prostate cancer risk [28–30]. In the present study our aim was to explore the effects of high consumption of meat, milk, and whole grain products in adolescence and midlife on breast cancer risk later in life, with a main emphasis on the adolescence period.

## Materials and methods

### Study population

We used data from the Age Gene Environment Susceptibility (AGES)—Reykjavik Cohort Study of the Icelandic Heart Association, a sub-cohort of the population based Reykjavik Study initiated in 1967 [31]. The AGES-Reykjavik Study examinations began in 2002 and at that time 11,549 Reykjavik Study cohort members were still alive. Thereof, 8,030 individuals were randomly invited to the study and of these, 5,764 individuals (thereof 3,326 women) participated between 2002–2006 (72% response rate). Extensive data were collected during clinical examinations, including information on food intake in adolescence, midlife and present old age. For our analysis we used data from the first clinical examination [32]. For this study, we excluded women who had been diagnosed with breast cancer prior to study entry (n = 196), leaving 3130 women in our study.

### Dietary assessment in early life and midlife

At study entry, the participants completed a food frequency questionnaire (FFQ) on diet in adolescence (between the ages of 14 to 19 years), in midlife (between the ages 40 to 50 years), and at study entry (late life). For this analysis we only use questions from the adolescent and midlife period.

The FFQ was especially designed for this project and provides information on frequency of intake of 10 common foods and food groups consumed in adolescence and 11 in midlife. For both adolescence and midlife, these food groups were meat (including salted and smoked meat), fish (including salted or smoked fish), fish liver oil, blood or liver sausage, rye bread, oatmeal, potatoes, milk and milk products, fruits and vegetables [33]. An additional question on consumption of whole wheat bread was included for the midlife period, but this type of bread was uncommon in the adolescent period. As previously stated, for this analysis our main focus is on meat, including salted and smoked meat, milk, rye bread, oatmeal and whole wheat bread. We have previously published analysis on fish and cod liver oil from this same cohort [27] while analysis on fruits and vegetables were not conducted due to very low consumption on a daily basis for both adolescence and midlife. However, all food groups, as described above, were included in the dietary pattern analysis (see supporting information).

Two separate questions were asked regarding meat consumption. One included total consumption of meat and ground meat as a meal (hereafter referred to as meat). The other question (included in total meat consumption) concerned intake of corned meat, corned meat sausage, or any kind of salted/smoked meat (hereafter referred to as salted or smoked meat). Information on milk consumption included frequency of intake of milk and milk products (hereafter referred to as milk). Rye bread consumption was assessed by one question on intake of rye bread and flatbread made of rye (hereafter referred to as rye bread). For midlife, the question on oatmeal also included muesli, but will be referred to as oatmeal in both periods.

For meat, milk, rye bread, oatmeal, and whole wheat bread, the frequency of consumption was classified into; 1) never, 2) less than once a week, 3) 1–2 times a week, 4) 3–4 times a week, 5) 5–6 times a week, 6) daily, and 7) more than once a day. For salted or smoked meat, the following response categories were: 1) never, 2) less than once a month, 3) 1–3 times a month, 4) 1–2 times a week 5) 3–6 times a week 6) daily or more often.

For both adolescence and midlife, meat consumption was divided into two categories. low (2 times or less per week) and high (3 times or more per week). Consumption of salted or smoked meat was also divided into two categories, with low intake defined as 3 times per month or less and high as once per week or more. Consumption of milk, rye bread and whole wheat bread (midlife only) was divided into two categories (less than daily and daily or more). Consumption of oatmeal was divided into low (4 times a week or less) and high (5 times a week or more).

The FFQ has been validated for midlife and late life [33, 34]. In short, midlife dietary habits were validated by comparing the results in the AGES-FFQ (n = 107) with detailed dietary data gathered from the same individuals 18–19 years previously in a National nutrition survey conducted in 1990. The main results were that the correlation coefficients for most of the food items were within an acceptable range [33].

### Covariate assessment

**Fig 1:**
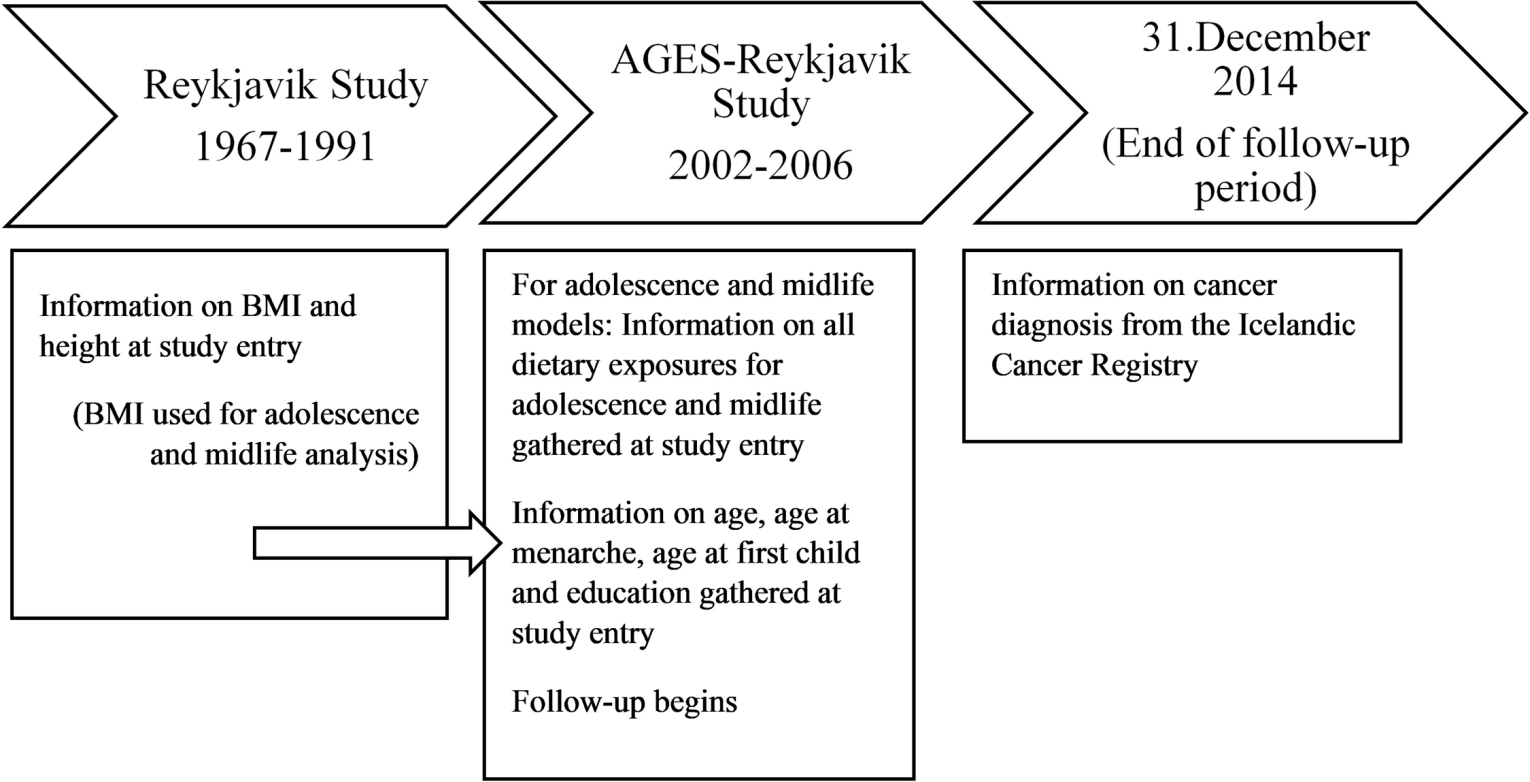
Timeline of examination points in the study.

Information on potential confounders was mainly retrieved from a lifestyle questionnaire, completed at entry to the AGES-Reykjavik Study. We collected information on age at entry to the study (continuous), age at menarche (continuous), family history of breast cancer (mother, sister and/or daughter ever diagnosed with breast cancer), education (primary, secondary, college/university), use of hormonal replacement therapy (never, ever), oral contraceptive (never, ever), use of alcohol in midlife (yes, no), and physical activity in adolescence and midlife (never/rarely, occasionally, moderately/often). We also retrieved information on ever being pregnant (y/n) and age at first birth (continuous), and combined this information into one variable (no births, age 24 and younger, 25 and older). From the Reykjavik Study we retrieved values on body mass index (BMI) and height from the midlife period (both continuous). Information on dietary covariates was retrieved from the AGES-FFQ (Fig 1).

Selected dietary covariates on concurrent consumption included, milk, meat, salted and smoked meat, oatmeal, and rye bread, depending on the exposure (see cut offs above). We also used information on fish consumption, (≤ 2 portions p/w vs. >2–4 portions p/w vs. > 4portions p/w) and on salted and smoked fish (3 times a month or less vs. once a week or more).

As residence dependent variance in food consumption existed during the adolescence period [5] we also added information on place of residence when growing up in our models (capital, coastal village, rural area, and combination of coastal village and rural area) collected in the Reykjavik Study.

### Ascertainment of outcome

We ascertained breast cancer diagnosis through the nationwide Icelandic Cancer Registry [35]. Information on cause of death was obtained from Directorate of Health. Due to the computerized national roster and unique personal identification numbers for each person, follow-up was virtually complete (99%) [35]. Participants were followed from the study entry (between 2002 and 2006) to diagnosis of breast cancer, death, or until the end of the observation period (December 31, 2014), whichever occurred first.

### Statistical analysis

For both adolescence and midlife, Cox proportional hazard regression models were used to calculate hazard ratio (HR) and 95% confidence interval (95% CI) for incident breast cancer. For all exposures in the adolescent period the first model was adjusted for age (as a continuous variable) at entry. Depending on exposure, all other food items under study (meat, salted and smoked meat, milk, rye bread, oatmeal) plus information on fish and salted and smoked fish were then added simultaneously to the second model (see cutoffs above in *covariates assessment*). Further adjustments were then made for education (3 categories), BMI (continuous) in midlife, age at first child birth (3 categories) and age at menarche (continuous).

After combining information on ever being pregnant and age at first birth in to one variable we had missing values for 211 women. The missing values were included in the multivariable analysis as a special category. We also replaced the 21 missing values for BMI with the mean BMI value of the participants of the study, or 25. The 177 missing values for age at menarche were replaced with the mean age of menarche in the cohort, or 14 years. We did not replace missing values for food items, neither for exposures or covariate variables.

For midlife, the same types of models were presented, except here midlife measures for all food consumption were used. Also, information on consumption of alcohol and whole wheat bread was added to the multivariate midlife model.

For both periods,further adjustment for family history of breast cancer, height, and physical activity, family history of breast cancer, hormonal replacement therapy or oral contraceptive did not change our risk estimates (data not shown) and were therefore not included in our final statistical models.

To assess the potential effects of longitudinal dietary habits on breast cancer risk we pooled consumption of each food item in adolescence and midlife into one variable with four categories; 1) low in both adolescence and midlife; 2) low in adolescence and high in midlife; 3) high in adolescence and low in midlife; and 4) high in both adolescence and midlife. For this analysis, the first category (low consumption in both periods) was used as a reference. Adjustments were made for same factors as described for the adolescent period. We also performed Spearman´s correlation test between consumption on food items under study in adolescence and midlife.

To further examine whether the association observed for rye bread and oatmeal persisted when other food items commonly consumed were included we also used principal component analysis to identify dietary patterns from the AGES-FFQ, including all dietary data available. This method is data driven and forms new linear factors (dietary patterns) by reducing data dimension and grouping correlated variables (food intake). For each pattern, a new variable is created, ranking participants on their adherence to that particular pattern [36]. Each variable/pattern was further divided into tertiles, or low, medium, and high adherence to each pattern. We used Cox proportional hazard regression to test association between adherence to adolescence and midlife patterns and breast cancer risk, using the lowest tertile as a reference. For both adolescence and midlife, adjustments were made for age at entry, BMI, education, age at menarche, and age at first child, using the same cut offs as previously described for individual exposures in adolescence and midlife. We also tested for trend in the hazard ratios for the first category, using polynomial contrasts (data shown in S1 and S2 Tables).

For all statistical analysis we used SPSS software, version 24.0 (SPSS Inc., Chicago, Illinois; www.spss.com) and R Core Team (2014). R: A language and environment for statistical computing. R Foundation for Statistical Computing, Vienna, Austria; (http://www.R-project.org/).

The study protocol was approved by the Icelandic Ethical Review Board and the Icelandic Data Protection Authority (VSN b2007120014/03-7).

## Results

The mean age at study entry was 77.0 years and standard deviation (SD) 6.0. The mean follow-up time was 8.8 years (SD = 3.1). During that time, 97 women were diagnosed with breast cancer, with mean age at diagnosis 81.4 years (SD = 6.5). The dietary analyses were based on participants who provided information on diet in midlife and adolescence when entering the AGES-Reykjavik cohort and were free of breast cancer.

### Diet in adolescence

Table 1 shows characteristics of the population that provided data on meat (n = 2,866), milk (n = 2,871), and rye bread (n = 2,858) consumption in adolescence. When compared with women with low consumption of meat in adolescence, women with high consumption of meat (3 times or more p/w) were older at study entry, less educated, and older when having their first child. They also consumed more salted or smoked meat and more salted and regular fish. Women with high milk consumption (daily or more often) had lower BMI in midlife, were more often raised in rural areas, and had more frequent consumption of cod liver oil, salted or smoked fish, total fish, meat, and oatmeal. When compared with less than daily rye bread consumption, women who consumed rye bread daily or more often were older when entering the study, had lower education level, were more commonly raised in rural areas, were older upon first birth, and less physically active. They also consumed fish more frequently, particularly salted or smoked fish as well as milk, oatmeal, and meat, including salted or smoked meat.

**Table 1:** Characteristics of female participants in the AGES–Reykjavik Study (2002–2006) according to consumption of rye bread, meat and milk in adolescence.

	Rye bread					Meat					Milk				
	N = 2858					N = 2866					N = 2871				
	Low		High			Low		High			Low		High		
	*Less than daily*		*Daily or more*			*2 times or less per week*		*3 times or more per week*			*Less than daily*		*Daily or more*		
	n = 1452		n = 1406		*P value*	n = 958		n = 1908		*P value*	n = 695		n = 2176		*P value*
**Age at study entry**															
Mean (SD)	75.2	(5.3)	77.8	(5.7)	0.001	76.1	(5.8)	76.6	(5.5)	0.001	76.3	(5.7)	76.5	(5.6)	0.549
Median	74		78			75		76			75		76		
Height (cm)*															
Mean (SD)	164.4	(5.2)	163.9	(5.5)	0.012	164.3	(5.4)	164.1	(5.4)	0.001	163.9	(5.1)	164.2	(5.5)	0.202
Median	164.5		164			164		164			164		164		
**BMI (kg/m**^**2**^)*															
Mean (SD)	24.9	(3.7)	25.0	(3.8)	0.520	24.8	(3.8)	25.0	(3.8)	0.001	25.3	(3.9)	24.8	(3.7)	0.010
Median	24		24.5			24		24			24.5		24		
**Education, n (%)**															
Primary	560	(39)	667	(47)	0.001	358	(37)	874	(46)	0.001	316	(46)	915	(42)	0.232
Secondary	665	(46)	572	(41)		446	(47)	792	(42)		292	(42)	951	(44)	
University/College	227	(15)	167	(12)		154	(16)	242	(12)		87	(13)	310	(14)	
**Birth cohort, n (%)**															
1908–1919	85	(6)	236	(17)	0.001	104	(11)	216	(11)	0.016	73	(11)	247	(11)	0.386
1920–1924	285	(20)	373	(26)		205	(21)	456	(24)		157	(23)	505	(23)	
1925–1929	470	(32)	435	(31)		283	(30)	621	(33)		209	(30)	699	(32)	
1930–1935	612	(42)	362	(26)		366	(38)	615	(32)		256	(37)	725	(33)	
**Location of first residence, n (%)**															
Reykjavik	603	(42)	458	(33)	0.001	351	(37)	712	(38)	0.001	283	(41)	783	(37)	0.001
Coastal village	515	(36)	417	(30)		267	(28)	666	(36)		270	(39)	664	(31)	
Rural area	259	(18)	450	(33)		277	(30)	437	(23)		113	(17)	601	(28)	
Combination of coastal village and rural area	53	(4)	50	(4)		45	(5)	58	(3)		18	(3)	86	(4)	
**Age at menarche, n (%)**															
≤ 13 y	669	(46)	609	(43)	0.147	447	(47)	836	(44)	0.147	309	(45)	978	(45)	0.824
≥ 14 y	782	(54)	794	(57)		510	(53)	1069	(56)		385	(55)	1195	(55)	
**Age at first birth, n (%)**															
No children	101	(7)	111	(8)	0.003	81	(8)	132	(7)	0.017	40	(6)	173	(8)	0.172
≤ 24 y	927	(64)	804	(57)		544	(57)	1197	(63)		441	(63)	1300	(60)	
≥ 25 y	404	(28)	472	(34)		317	(33)	557	(29)		205	(30)	673	(31)	
**Physical activity**															
Never	600	(44)	685	(52)	0.001	405	(46)	881	(49)	0.179	310	(47)	977	(48)	0.499
Rarely/occasionally	335	(25)	242	(19)		202	(23)	376	(21)		153	(23)	426	(21)	
Moderate/high	426	(31)	377	(29)		281	(32)	526	(30)		194	(30)	615	(31)	
**Family history of breast cancer**															
Yes	235	(16)	238	(17)	0.593	147	(15)	330	(17)	0.186	121	(17)	355	(16)	0.499
**High intake of other food groups**															
**Fish** (> 4 portions p/w)	532	(37)	606	(43)	0.001	186	(19)	956	(50)	0.001	256	(37)	887	(41)	0.045
**Salted fish** (once p/w or more)	600	(42)	861	(62)	0.001	450	(47)	1008	(53)	0.002	255	(37)	1210	(56)	0.001
**Oatmeal** (≥ 5 times p/w)	377	(26)	686	(49)	0.001	353	(37)	710	(38)	0.840	132	(19)	932	(43)	0.001
**Salted or smoked meat** (once p/w or more)	356	(25)	575	(41)	0.001	334	(35)	595	(31)	0.053	146	(21)	783	(36)	0.001
**Milk (**daily or more)	974	(67)	1185	(84)	0.001	733	(77)	1432	(75)	0.328					
**Meat** ≥ 3 times p/w)	944	(65)	953	(68)	0.122						473	(68)	1432	(66)	0.328

Abbreviations: SD, standard deviation.

*Measured in midlife—at entry to the Reykjavik Study

P values are based on Chi-square tests, except for age at entry, height and BMI, where One-Way ANOVA test was used

Table 2 presents hazard ratios, with 95% CI, for breast cancer and diet in adolescence. No statistically significant association was found for meat, salted and smoked meat and milk consumption. For whole grain products, we found a positive association between high consumption of rye bread (daily or more often) in adolescence and breast cancer risk (HR 1.7, 95% CI 1.1–2.6), when compared with lower consumption (less than daily). No significant association was observed for oatmeal consumption. When adding early life residence to the multivariate model the risk estimates for high rye bread consumption increased (HR 1.8, 95% CI 1.2–2.9). No difference was observed for other estimates.

**Table 2:** Multivariate analysis of breast cancer risk by meat, milk and whole grain intake in adolescence.

	Meat		Salted or smoked meat		Milk		Rye bread		Oatmeal	
	N = 2866		N = 2862		N = 2871		N = 2858		N = 2856	
	Low	High	Low	High	Low	High	Low	High	Low	High
	*2 times or less p/w*	*3 times or more p/w*	*3 times a month or less*	*Once p/w or more*	*Less than daily*	*Daily* *or more*	*Less than daily*	*Daily or more*	*4 times or less p/w*	*5 times or more p/w*
	n = 958	n = 1908	n = 1930	n = 932	n = 695	n = 2176	n = 1452	n = 1406	n = 1791	n = 1065
**Breast cancer** **(%)**	28 (2.9)	68 (3.6)	61 (3.2)	36 (3.9)	29 (4.2)	68 (3.1)	41 (2.8)	55 (3.9)	68 (3.8)	29 (2.7)
**Age adjusted HR**	1.0	1.2	1.0	1.3	1.0	0.8	1.0	1.5	1.0	0.7
**(95% CI)**	(ref.)	(0.8–1.9)	(ref.)	(0.8–1.9)	(ref.)	(0.50–1.2)	(ref.)	(1.0–2.2)	(ref.)	(0.5–1.2)
**Multivariate** HR*	1.0	1.3	1.0	1.4	1.0	0.7	1.0	1.7	1.0	0.7
**(95% CI)**	(ref.)	(0.8–2.0)	(ref.)	(0.9–2.2)	(ref.)	(0.4–1.1)	(ref.)	(1.1–2.6)	(ref.)	(0.5–1.2)

**Abbreviations:** CI, confidence interval; HR, hazard ratio: p/w, per week

*Multivariate HR; adjusted for age at entry, education, body mass index in midlife, age at first child and age at menarche. All food items under study (meat, salted and smoked meat, milk, rye bread, oatmeal) plus information on fish and salted and smoked fish were then added simultaneously to the multivariate model. The multivariate analysis included 2810 women, thereof 95 with breast cancer.

We further divided rye bread consumption into three groups (two times or less per week, 3–6 times per week, and daily or more) and explored the association with breast cancer risk, where we found a significant trend across the groups, HR 1.0 (95% CI 0.5–2.0) and HR 1.7 (95% CI 0.9–3.2), respectively (P_trend_ = 0.043).

### Midlife diet

For midlife (Table 3), no significant association was observed for meat and milk consumption, although a marginally positive association was observed for high consumption (once per week or more) of salted or smoked meat (HR 1.6, 95% CI 1.0–2.6) compared with women with low consumption (3 times a month or less). For whole grain products, no association was observed for whole wheat bread while a statistically significant positive association was observed for high consumption (daily or more) of rye bread (HR 1.8, 95% CI 1.1–2.9) when compared with lower consumption (less than daily). No significant association was observed for oatmeal. No difference was observed for any of the risk estimates when early life residence was added to the model.

**Table 3:** Multivariate analysis of breast cancer risk by meat, milk and whole grain intake in midlife.

	Meat		Salted or smoked meat		Milk		Rye bread		Oatmeal		Whole wheat bread	
	N = 2871		N = 2864		N = 2864		N = 2875		N = 2768		N = 2865	
	Low	High	Low	High	Low	High	Low	High	Low	High	Low	High
	*2 times or less p/w*	*3 times or more p/w*	*3 times a month or less*	*Once p/w or more*	*Less than daily*	*Daily or more*	*Less than daily*	*Daily or more*	*4 times or less p/w*	*5 times or more p/w*	*Less than daily*	*Daily or more*
	n = 1203	n = 1668	n = 2210	n = 654	n = 1247	n = 1617	n = 1918	n = 957	n = 2094	n = 674	n = 1238	n = 1627
**Breast cancer** **(%)**	41 (3.4)	56 (3.4)	71 (3.2)	26 (4.0)	41 (3.2)	56 (3.5)	57 (3.0)	40 (4.2)	81 (3.7)	16 (2.3)	44 (3.6)	53 (3.3)
**Age adjusted** **HR**	1.0	1.0	1.0	1.3	1.0	1.1	1.0	1.5	1.0	0.6	1.0	0.9
**(95% CI)**	(ref.)	(0.6–1.5)	(ref.)	(0.8–2.1)	(ref.)	(0.7–1.6)	(ref.)	(1.0–2.3)	(ref.)	(0.4–1.1)	(ref.)	(0.6–1.4)
**Multivariate** HR*	1.00	1.0	1.0	1.6	1.0	1.1	1.0	1.8	1.0	0.6	1.0	0.8
**(95% CI)**	(ref.)	(0.6–1.4)	(ref.)	(1.0–2.6)	(ref.)	(0.7–1.7)	(ref.)	(1.1–2.9)	(ref.)	(0.4–1.1)	(ref.)	(0.5–1.3)

Abbreviations: CI, confidence interval; HR, hazard ratio.

*Multivariate HR; adjusted for age at entry, education, body mass index in midlife, alcohol consumption in midlife, age at first child and age at menarche. All food items under study (meat, salted and smoked meat, milk, rye bread, oatmeal and whole wheat bread) plus information on fish and salted and smoked fish were then added simultaneously to the multivariate model. The multivariate analysis included 2813 women, thereof 97 with breast cancer.

Rye bread consumption in midlife was further divided into three groups (two times or less per week, 3–6 times per week, and daily or more). Compared with the group with the lowest consumption, the risk estimates for 3–6 times per week and daily or more were 1.4 (95% CI 0.8–2.4) and 2.3 (95% CI 1.3–4.1), respectively (P_trend_ < 0.01).

### Long term consumption

Table 4 presents Spearman’s correlation between dietary habits in adolescence and midlife. The strongest correlation was found for rye bread consumption (ρ = 0.50, *P* = 0.001) and the lowest for meat consumption (ρ = -0.19, *P* = 0.001).

**Table 4:** Dietary habits among participants through different time periods.

	Adolescence	Midlife	Spearmans´s ρ	*P*
	**n (%)**	**n (%)**		
**Rye bread**			0.50	0.001
**Less than daily**	1452 (51)	1918 (67)		
**Daily or more**	1406 (49)	957 (31)		
**Milk and milk products**			0.44	0.001
**Less than daily**	695 (24)	1247 (44)		
**Daily or more**	2176 (76)	1617 (56)		
**Meat**			-0.19	0.001
**2 times or less p/w**	958 (33)	1203 (42)		
**3 times or more p/w**	1908 (67)	1668 (58)		
**Salted or smoked meat**			0.36	0.001
**3 times a month or less**	1930 (67)	2210 (77)		
**Once or more p/w**	932 (33)	654 (23)		
**Oatmeal**			0.38	0.001
**4 times or less p/w**	1791 (63)	2715 (76)		
**5 times or more p/w**	1065 (37)	690 (24)		
**Vegetables**			0.38	0.001
**Never**	585 (20)	163 (6)		
**6 times p/w or less**	2175 (76)	2845 (87)		
**Daily**	105 (4)	213 (7)		
**Fruit**			0.24	0.001
**Never**	1013 (36)	114 (4)		
**6 times p/w or less**	1784 (62)	2501 (87)		
**Daily**	68 (2)	256 (9)		

Table 5 presents long term consumption for meat, salted or smoked meat, milk, rye bread, and oatmeal with low consumption in adolescence and midlife as a reference category. A positive association was observed for high rye bread consumption in adolescence and midlife (HR 2.1, 95% CI 1.2–3.5). An inverse association was observed between breast cancer risk and high consumption of oatmeal in both adolescence and midlife (HR 0.4, 95% CI 0.2–0.9) (P_trend_ = 0.07). No association was observed between breast cancer and meat, salted or smoked meat and milk.

**Table 5:** Breast cancer risk by longitudinal intake of meat, milk and whole grains.

	Adolescence	Midlife	N	Breast cancer (%)	Age adjusted HR (95% CI)	Multivariate HR (95% CI)*
**Meat**						
	Low	Low	275	5 (1.8)	1.0, (ref.)	1.0, (ref.)
	Low	High	681	23 (3.4)	1.8 (0.7–4.8)	1.9 (0.7–5.2.1)
	High	Low	921	35 (3.8)	2.1 (0.8–5.4)	2.0 (0.9–5.6)
	High	High	980	33 (3.4)	1.8 (0.7–4.7)	2.0 (0.8–5.3)
**Salted or smoked meat**						
	Low	Low	1682	52 (3.1)	1.0, (ref.)	1.0, (ref.)
	Low	High	239	9 (3.8)	1.3 (0.6–2.6)	1.3 (0.6–2.6)
	High	Low	517	19 (3.7)	1.2 (0.7–2.1)	1.4 (0.8–2.5)
	High	High	412	17 (4.1)	1.4 (0.8–2.5)	1.6 (0.8–2.9)
**Milk**						
	Low	Low	567	24 (4.2)	1.0, (ref.)	1.0, (ref.)
	Low	High	122	5 (4.1)	1.0 (0.4–2.5)	0.9 (0.3–2.4)
	High	Low	674	17 (2.5)	0.6 (0.3–1.1)	0.6 (0.3–1.1)
	High	High	1492	51 (3.4)	0.8 (0.5–1.3)	0.8 (0.5–1.3)
**Rye bread**						
	Low	Low	1304	34 (2.6)	1.0, (ref.)	1.0, (ref.)
	Low	High	145	7 (4.8)	2.1 (0.9–4.8)	2.4 (1.0–5.4)
	High	Low	597	22 (3.7)	1.5 (0.9–2.6)	1.7 (1.0–3.0)
	High	High	807	33 (4.1)	1.7 (1.0–2.8)	2.1 (1.2–3.5)
**Oatmeal**						
	Low	Low	1577	59 (3.7)	1.0, (ref.)	1.0, (ref.)
	Low	High	206	9 (4.4)	1.1 (0.6–2.3)	1.0 (0.5–2.1)
	High	Low	584	22 (3.8)	1.0 (0.6–1.7)	1.0 (0.6–1.7)
	High	High	478	7 (1.5)	0.4 (0.2–0.9)	0.4 (0.2–0.9)

Abbreviations: CI, confidence interval; HR, hazard ratio.

For meat; low stands for 2 times or less p/w; high for 3 times or more p/w.

For salted or smoked meat; low stands for 3 times a month or less; high stands for once p/w or more.

For oatmeal; low stands for 4 times and less p/w; high stands for 5 times or more p/w.

For milk and rye bread low stands for less than daily; high stands for daily or more.

*Multivariate HR; adjusted for age at entry, education, body mass index in midlife, age at first child and age at menarche. With the exception of the food item under study each time—all other food items in adolescence (meat, salted and smoked meat, milk, rye bread, oatmeal) plus information on fish and salted and smoked fish in adolescence were then added simultaneously to the multivariate model.

All multivariate analysis includes 95 breast cancer events.

### Supplementary material—dietary pattern

Four dietary patterns were extracted for the adolescent period. Factor loading coefficients for those patterns are presented in S1 Table. One pattern containing rye bread in addition to blood liver sausage, salted meat, salted fish, and oatmeal represents traditional Icelandic diet in the earlier half of the 20^th^ century. High adherence to this pattern was not significantly associated with breast cancer risk (HR 1.3 95% CI 0.7–2.1). Borderline inverse association was observed for the highest adherence to a pattern high in consumption of fish, blood/liver sausage, oatmeal, fish oil, and milk in adolescence (HR 0.6, 95% CI 0.4–1.0) (P_trend_ = 0.049) (S2 Table).

Four dietary patterns were also extracted for the midlife period. No association was observed for any of those patterns, including the dietary pattern including rye bread consumption (data not shown).

## Discussion

In this population based cohort, daily consumption of rye bread during both adolescence and midlife was positively associated with breast cancer. Conversely, we found reduced risk for breast cancer among women who consumed oatmeal persistently both in adolescence and midlife. However, no dietary pattern in either adolescence or midlife that included rye bread was significantly associated with breast cancer while a pattern in adolescence that represented fish, blood/liver sausage, oatmeal, fish oil, and milk seemed to be protective for breast cancer. We also observed borderline risk of breast cancer with high midlife consumption of salted or smoked meat. No association was found between high milk intake and breast cancer risk in either period.

To the best of our knowledge, no study has specifically addressed adolescent consumption of rye bread, a common whole grain product in the Nordic countries, in relation to late-life breast cancer. A few studies have analyzed total adolescent consumption of lignans, a common phytoestrogen in whole grain cereals—such as rye, wheat, oats, and barley—but also in legumes, oilseeds, and various fruits and vegetables [37]. A Canadian case control study found that high adolescent intake of lignans reduced the risk of breast cancer. However, although rye bread was included in the diet assessment of the study, it was not commonly consumed and these results can also be confounded by other healthy eating habits [20]. Results from the Nurses' Health Study suggest that high consumption of whole grains in adolescence can reduce the risk of pre-menopausal breast cancer [22]. However, the main difference between this study and ours was rye bread does not seem to be included in total wholegrain consumption in the Nurse´s Health Study questionnaire. Also, the women in our cohort were all post-menopausal with a high mean age at diagnosis, and therefore possibly different carcinogenic process [38].

The few studies on total whole grain consumption in adults and breast cancer have either suggested a protective [23, 24] or no association [25, 26]. The major difference between most of these and the present study is that we disentangled whole grain consumption into rye, whole wheat bread and oatmeal. Different types of whole grains contain dissimilar bioactive compounds [7] which may act differently on breast cancer genesis.

A Danish study [25] reported no association between neither total adult consumption of whole grains nor when analyzed separately (rye bread, whole grain bread, and oatmeal) with breast cancer. However, the women in the Danish cohort were younger at study entry and at diagnosis (average age 56 years) than participants in the AGES-Reykjavik Study (average age 77 years). This might reflect a previously reported difference in characteristics of diagnosed breast tumors in older women (over 70) compared with those in younger women [38]. Indeed, we found no association for rye bread when a separate analysis was made for women who were already diagnosed with breast cancer at study entry (mean age at diagnosis 64 years, data not shown).

Prolonged exposure to rye bread might therefore possibly explain the observed risk found in our cohort. Rye bread is rich in lignans, which can be converted into enterolignans by the gut microbiome. Enterolignans have been suggested to enhance breast cancer risk reduction [7, 39, 40]. However, they can also express weak estrogenic affinity and can act both as agonists or antagonists in breast tumors. although this mechanism is considered complex [7, 41–43]. Nevertheless, longitudinal exposure to estrogens, exemplified by early menarche, late menopause, and use of hormonal replacement therapy, are considered as a major risk factors for breast cancer [44]. Therefore, it may be hypothesized that long term exposure to phytoestrogens via rye bread consumption may have a similar effect. Indeed, our analysis on dietary habits through different time-periods shows that the highest correlation between consumption in adolescence and midlife was found for rye bread. The rye grain also has some other bioactive compounds of unknown concentration [45] that may be of significance in this context. Also, common toppings for rye bread, like smoked meat, may contain other potentially carcinogenic compounds. Furthermore, potentially carcinogenic compounds could be formed when the old-style rye flatbread, a traditional Icelandic bread included in the question on rye bread, is baked or charred directly on a hot plate. Thus, these data point towards carcinogenic effects of some compounds in the rye bread or its cooking method. However, no significant results were observed for any dietary pattern that included high rye bread consumption, neither in adolescence nor the midlife period, suggesting that rye bread in the total diet might not be of major concern for cancer risk.

In contrast to our results on rye bread, frequent long-term consumption of oatmeal was found to be protective against breast cancer. Similar to rye, yet containing only half the amount of phytoestrogen [7], oatmeal is rich in fiber, which is thought to reduce breast cancer risk via multiple pathways [46–48]. Indeed, two studies on fiber intake in adolescence and early adulthood found an inverse association with breast cancer [19, 49]. However, when analyzed separately, only fiber from fruit and vegetables was protective effects against breast cancer in one study [19] whereas the main sources of fiber in the other study were not clear [49]. Although we cannot exclude the influence of fiber to be responsible for our beneficial results, oatmeal also contains multiple bioactive compounds, including the polysaccharide beta-glucan, which is proposed to have some anticancer properties. However, data on this association is still limited [50, 51]. The inclusion of muesli as part of the question on oatmeal might also act as proxy for consumption of other healthy food items. This is further supported by the borderline risk reduction for breast cancer found with high adherence to a dietary pattern in adolescence that included oatmeal, fish, fish oil, milk, and blood- and liver sausage. This further indicates possible anticancer properties of oatmeal and possible other food items in that particular dietary pattern.

A major strength of our study is the prospective design and the well-established population-based AGES cohort with its extensive covariate information. The record linkage to the nationwide Cancer Registry of Iceland ensures detailed and valid assessment of the outcome with a virtually complete follow-up [35], where all participants had equal access to the public health care system at study entry. Also, the especially designed validated FFQ used for assessment of food consumption in adolescence and midlife additionally gives a rare opportunity for evaluation of longitudinal consumption in relation to cancer diagnosis. However, the FFQ used has only crude information on quantity of food items consumed and we are not able to adjust for cooking methods, single nutrients, total intake of fat, fiber, and energy and we do not have information on types or quantities of condiments. Also, the dietary data are retrospective in nature and there is always risk of non-differential recall bias when dietary habits 40–50 years earlier are assessed [52]. Validation for the adolescent period is not possible. However, food-related memory from childhood to four decades later have been found to be accurate as food-related memory of current diet, especially for food items eaten rarely or daily [53]. Indeed, our data somewhat represents the residency based difference that existed in Iceland and it should be noted that diet in Iceland during the adolescent period was quite simple, with very few food items available and little day to day variation [5],making the recall easier. The results of the validation study for midlife FFQ did however find low correlation for rye bread consumption (r = 0.507, p = 0.066). Furthermore, as our results are based on older women diagnosed with breast cancer, we cannot draw any firm conclusion for women diagnosed earlier in life. We also do not have information on age of menopause or BMI for adolescence. Finally, we cannot exclude any unmeasured confounding affecting our results or that our findings are due to chance.

## Conclusion

In conclusion, our results suggest that rye bread consumption in both adolescence and midlife is associated with increased risk of breast cancer diagnosed late in life. Conversely, persistent high consumption of oatmeal in adolescence and midlife was associated with decreased risk. Collectively, these data suggest that dietary exposure during both adolescence and midlife period might be critical for breast cancer development in older women. These associations need to be confirmed in future studies, and our findings also call for further studies on potential mechanisms involved.

## Supporting information

S1 Table Factor loading coefficient for dietary pattern in adolescence. (DOCX)

S2 Table Hazard ratios (HR) and 95% confidence intervals (95% CI) for breast cancer diagnoses by tertiles of dietary pattern in adolescence. (DOCX)
