# Correction: Dietary habits in adolescence and midlife and risk of breast cancer in older women

**DOI:** 10.1371/journal.pone.0206026

**Published:** 2018-10-15

**Authors:** Alfheidur Haraldsdottir, Johanna E. Torfadottir, Unnur A. Valdimarsdottir, Hans-Olov Adami, Thor Aspelund, Laufey Tryggvadottir, Marianna Thordardottir, Bryndis E. Birgisdottir, Tamara B. Harris, Lenore J. Launer, Vilmundur Gudnason, Laufey Steingrimsdottir

## Notice of republication

The original S1 File was published in error. The file was removed from the HTML and PDF versions of this article on October 5, 2018. The Data Availability statement has also been updated to reflect this change. Please download this article again to view the correct version.
