# Supplementary material for: Dietary habits in adolescence and midlife and risk of breast cancer in older women
Source: PLoS One. 2018 May 30;13(5):e0198017. doi: 10.1371/journal.pone.0198017 (PMC5976175; doi:10.1371/journal.pone.0198017)
Supplement: S1 Table — (DOCX) [file pone.0198017.s001.docx]

**Supplementary table 1.** Factor loading coefficient for dietary pattern in adolescence

|  | **Factor loading coefficient ^a, b^** |
| --- | --- |
| ***Pattern 1*** |  |
| Blood/liver sausage | 0.60 |
| Salted meat | 0.79 |
| Salted fish | 0.73 |
| Rye bread | 0.46 |
| Oatmeal | 0.38 |
| Milk | 0.40 |
| ***Pattern 2*** |  |
| Fish meal | -0.31 |
| Fish as a side | 0.64 |
| Fruit | 0.76 |
| Vegetables | 0.76 |
| ***Pattern 3*** |  |
| Fish meal | 0.34 |
| Blood/liver sausage | 0.33 |
| Oatmeal | 0.63 |
| Fish oil | 0.74 |
| Milk | 0.31 |
| ***Pattern 4*** |  |
| Meat | 0.73 |
| Fish meal | 0.58 |
| Potatoes | 0.48 |

^a^ Factor loadings are correlation coefficients between

food groups and the extracted factor.
^b^ Food groups with factor loading between 0.30 and

-0.30 are not listed.
