# Supplementary material for: Dietary habits in adolescence and midlife and risk of breast cancer in older women
Source: PLoS One. 2018 May 30;13(5):e0198017. doi: 10.1371/journal.pone.0198017 (PMC5976175; doi:10.1371/journal.pone.0198017)
Supplement: S2 Table — (DOCX) [file pone.0198017.s002.docx]

**Supplementary table 2.** Hazard ratios (HR) and 95% confidence intervals (95% CI) for breast cancer diagnoses by tertiles of dietary pattern in adolescence

|  | **BC (%)** | **Age adjusted HR**  **(95% CI)** | **Multivariate HR**  **(95% CI)*** |
| --- | --- | --- | --- |
| ***Pattern 1 (n = 2776)*** | |  |  |
| Low adherence | 26 (2.8) | 1.0 (ref.) | 1.0 (ref.) |
| Medium adherence | 38 (4.1) | 1.5 (0.9 – 2.5) | 1.5 (0.9 – 2.5) |
| High adherence | 31 (3.3) | 1.3 (0.8 – 2.1) | 1.3 (0.7 – 2.1) |
| *P _linear trend_* |  | *0.103* | *0.297* |
| ***Pattern 2 (n =2776)*** | |  |  |
| Low adherence | 29 (3.1) | 1.0 (ref.) | 1.00 (ref.) |
| Medium adherence | 35 (3.8) | 1.2 (0.8 – 2.0) | 1.3 (0.8 – 2.2) |
| High adherence | 31 (3.4) | 1.1 (0.7 – 1.8) | 1.2 (0.7 – 2.1) |
| *P _linear trend_* |  | *0.748* | *0.471* |
| ***Pattern 3 (n = 2775)*** | |  |  |
| Low adherence | 40 (4.3) | 1.0 (ref.) | 1.0 (ref.) |
| Medium adherence | 31 (3.4) | 0.8 (0.5 – 1.3) | 0.8 (0.5 – 1.3) |
| High adherence | 24 (2.6) | 0.6 (0.5 – 1.0) | 0.6 (0.4 – 1.0) |
| *P _linear trend_* |  | *0.066* | *0.049* |
| ***Pattern 4 (n = 2777)*** | |  |  |
| Low adherence | 32 (3.5) | 1.0 (ref.) | 1.0 (ref.) |
| Medium adherence | 26 (2.8) | 0.8 (0.5 – 1.4) | 0.8 (0.5 – 1.3) |
| High adherence | 37 (4.0) | 1.2 (0.7 – 1.9) | 1.2 (0.7 – 1.) |
| *P _linear trend_* |  | *0.529* | *0.558* |

Low adherence stands for tertile 1; Medium adherence stands for tertile 2; High adherence stands for tertile 3.

*Multivariate HR: Adjusted for age at entry, BMI, education, age at menarche and age at first birth. 95 breast cancer events were included in the multivariate analysis.
